# A New Test of Attention in Listening (TAIL) Predicts Auditory Performance

**DOI:** 10.1371/journal.pone.0053502

**Published:** 2012-12-31

**Authors:** Yu-Xuan Zhang, Johanna G. Barry, David R. Moore, Sygal Amitay

**Affiliations:** 1 Medical Research Council - Institute of Hearing Research, Nottingham, United Kingdom; 2 National Key Laboratory of Cognitive Neuroscience and Learning, Beijing Normal University, Beijing, China; UNLV, United States of America

## Abstract

Attention modulates auditory perception, but there are currently no simple tests that specifically quantify this modulation. To fill the gap, we developed a new, easy-to-use test of attention in listening (TAIL) based on reaction time. On each trial, two clearly audible tones were presented sequentially, either at the same or different ears. The frequency of the tones was also either the same or different (by at least two critical bands). When the task required same/different frequency judgments, presentation at the same ear significantly speeded responses and reduced errors. A same/different ear (location) judgment was likewise facilitated by keeping tone frequency constant. Perception was thus influenced by involuntary orienting of attention along the task-irrelevant dimension. When information in the two stimulus dimensions were congruent (same-frequency same-ear, or different-frequency different-ear), response was faster and more accurate than when they were incongruent (same-frequency different-ear, or different-frequency same-ear), suggesting the involvement of executive control to resolve conflicts. In total, the TAIL yielded five independent outcome measures: (1) baseline reaction time, indicating information processing efficiency, (2) involuntary orienting of attention to frequency and (3) location, and (4) conflict resolution for frequency and (5) location. Processing efficiency and conflict resolution accounted for up to 45% of individual variances in the low- and high-threshold variants of three psychoacoustic tasks assessing temporal and spectral processing. Involuntary orientation of attention to the irrelevant dimension did not correlate with perceptual performance on these tasks. Given that TAIL measures are unlikely to be limited by perceptual sensitivity, we suggest that the correlations reflect modulation of perceptual performance by attention. The TAIL thus has the power to identify and separate contributions of different components of attention to auditory perception.

## Introduction

Auditory performance is determined by interactions of auditory sensation with attention, memory, vision, emotion and a variety of other, lesser influences [1], [2], [3], [4]. Attention and memory have received much recent interest because they are particularly important for the assessment and rehabilitation of hearing difficulties [5], [6], [7]. The influence of attention has typically been demonstrated by examining how directing attention to and away from the target stimuli or stimulus features alters psychophysical (e.g., the dichotic listening paradigm; [8]) or physiological (e.g., hemodynamic signals [9], neuromagnetic fields [10]) measures of sound perception. Our goal here was to develop a behavioral test of auditory attention that can be used to identify and quantify the contribution of attention to auditory performance.

Attention has been studied under a wide range of cognitive conditions and has so far eluded a consensual definition after over a century’s documented investigation [11]. At the core of most attention phenomena is the concept of selection: a subset of the available stimulus pool (including internal stimuli such as thoughts and memories) is examined more closely than and at the expense of others [12]. Most perceptual tasks also involve judgments based on a subset of stimuli or stimulus features (task relevant dimensions) among all that are present. For example, tone frequency discrimination requires judgments to be made based on tone pitch. Other aspects of the stimuli including level, duration, and location are not useful for successful task performance and are regarded as task irrelevant dimensions. In everyday life, we are constantly assigning and switching listening priority between different sound sources or features (e.g., voices [13]). Thus, the ability to select and focus on task relevant dimensions may contribute significantly to perceptual performance in addition to perceptual acuity. We developed a Test of Attention in Listening (TAIL) as the first step towards identifying and quantifying such contributions.

**Figure 1:**
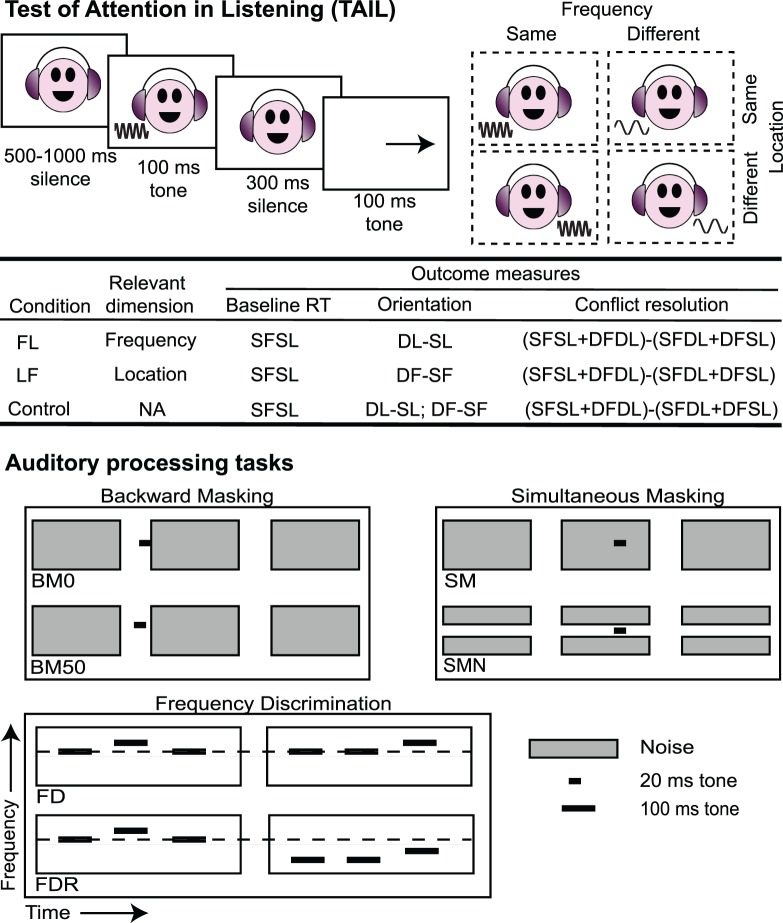
Schematic illustration of the Test of Attention in Listening (TAIL) and auditory processing tasks. For TAIL, Backward and Simultaneous Masking, one example trial of stimulus presentation is plotted. For Frequency Discrimination, two trials are plotted. Abbreviations: In TAIL, F stands for frequency, L for location, S for same, and D for different. In auditory processing tasks, BM0 is Backward Masking with no silence gap between the target tone and the noise masker, BM50 is Backward Masking with a 50-ms silence gap between the target tone and the noise masker, SM is Simultaneous Masking with no spectral notch around the target tone, SMN is Simultaneous Masking with a spectral notch around the target tone, FD is Frequency Discrimination with a fixed standard tone, and FDR is Frequency Discrimination with a roving standard tone.

TAIL measures the ability to focus selectively on a task relevant dimension and ignore information from task irrelevant dimensions using reaction time (RT) as the primary performance measure. In each trial, two clearly audible tones are presented sequentially (Fig. 1). The listener is asked to indicate whether the two tones are the same or different along one of two dimensions (frequency or location) as accurately and as quickly as possible. The other dimension is also systematically manipulated to serve as the distracting dimension. Highly distinctive variants of each stimulus dimension are used to avoid confound with perceptual difficulty. Other task-irrelevant dimensions (stimulus duration and level) are roved.

The design and interpretation of the TAIL were informed by and are consistent with the principles of attention embodied in the influential Attention System view [14], [15] and the Load Theory of attention [16]. The Attention System view suggests that attention is subserved by a system of brain networks that is neuroanatomically separate from the information processing system (including stimulus encoding, analyzing, and decision making) and that this attention system consists of three separable networks carrying out, respectively, the functions of maintaining vigilance, orienting attention, and executive control. According to this view, attention in the TAIL is directed (‘oriented’) to the task relevant dimension via the orienting attention network. According to the Load Theory, under such a low perceptual load (one tone at a time), perceptual capacity is not exhausted and attention will “spill over” to task irrelevant information, allowing further processing of that information. Load Theory thus predicts involuntary orientation of attention to the distracting dimension in proportion to perceptual capacity spared from relevant information processing. In addition, perceptual processing of information in the distracting dimension may present conflicts to decision making, as the same/different relationship in the distracting dimension may be incongruent with that in the task relevant dimension. Resolution of response conflicts would be indexed by performance deterioration in the incongruent relative to the congruent case. Conflict resolution is typically used as a measure of executive control (e.g., the 'Stroop task' [17], [18]). Finally, when the tones are the same in both dimensions (without distracting or conflicting information), RTs provide a ‘baseline’ measure of information processing under minimum attention control.

We examined attention contribution to auditory performance by comparing RT measures of TAIL with threshold measures on three psychoacoustic tasks emphasizing spectral and temporal processing [5], [19]: tone Frequency Discrimination, Backward Masking, and Simultaneous Masking. These tasks are recommended measures of ‘auditory processing’ (American Academy of Audiology, 2010) but may still be strongly influenced by attention skills [5]. For each task, we included a more demanding, high-threshold variant and a less demanding, low-threshold variant, in both of which threshold was assessed at 79% correct performance (Fig. 1). The stimuli used in these tasks were very simple, consisting of a single tone or a tone with a band-passed noise in each observation interval. Our current understanding of these tasks is not sufficient to make specific predictions about which attention components of the TAIL will contribute to performance on each task. However, some general trends can be predicted. First, these tasks showed differential activation of non-sensory cortical regions [20], [21]. For example, Backward Masking produced greater activity than Simultaneous Masking in anterior cingulate cortex [21], a brain region critical for conflict monitoring and executive control [14]. Thus, we expected differential patterns of attention contribution across the tasks. Second, as the simple stimuli are unlikely to exhaust perceptual capacity, we did not expect significant contribution of involuntary orientation. Third, and most critically, as the role of executive control is to facilitate focused attention onto the target information [14], [22], we predicted that conflict resolution would more likely contribute to the high-threshold task variants in which the challenge of separating task relevant information from irrelevant information is greater. Performance on the lower threshold variant, in contrast, would more likely reflect the efficiency of the information processing systems themselves.

## Materials and Methods

### Ethics Statement

The research protocol was approved by the Nottingham University Hospitals Research Ethics Committee. All of the participants gave informed written consent and received an inconvenience allowance for their participation.

### Participants

Nineteen volunteers between the age of 18 and 36 years (mean of 26 years, 9 females) were recruited from the University of Nottingham campus and nearby neighborhoods. All of the volunteers had 20 dB HL or better hearing thresholds for tones between 500 and 6000 Hz, bilaterally.

### Equipment

All testing was conducted in a sound attenuated booth on a PC, with all sounds delivered via circumaural headphones (Sennheiser HD 25). A USB-interfaced button box made in-house was used for response. The testing was fully automatic, with instructions displayed on the screen at the beginning of each block of trials.

### Task and Stimuli

**TAIL.** TAIL was run in three conditions, in all of which tone frequency (F) and location (L; ear at which the tone was presented) were systematically manipulated (Fig. 1). In the FL condition, frequency was the task-relevant dimension and location was the distracting dimension. Listeners were asked to press one of two buttons as accurately and as quickly as possible to indicate whether the two tones were the same or different in pitch. In the LF condition, location was the relevant and frequency the irrelevant dimension. Listeners chose whether the two tones were presented at the same or different ears. In the Control condition, neither frequency nor location was task relevant. Listeners were asked simply to press any button as soon as they heard the second tone. In all conditions, tone level was roved between 70 to 85 dB SPL and tone duration was roved between 100 and 300 ms. The silent gap between the two tones was fixed at 300 ms. Before the experiment, we ran pilot studies to ensure that variations within the roved ranges had no impact on the attention measures derived.

Four blocks of 40 trials were run for each condition, with the order of conditions randomized across listeners. Condition was switched between blocks and the corresponding instruction was displayed on the screen at the beginning of each block. Before the first block of each condition, a demo of 5 trials was used to familiarize the participants with the task. Each block of trials followed a two (same and different frequency) by two (same and different location) design. Tone frequencies were drawn randomly from the range 476–6188 Hz, with the constraint that the spectral gap between any two tones was at least 2.1 equivalent rectangular bandwidths (ERBs; [23]). This gap was well above the frequency discrimination thresholds of all of the participants and was intended to avoid perceptual confusion. The total twelve blocks of TAIL took about 20 minutes to complete.

Reaction times (RTs) on correct trials were used as the primary performance measure. RTs longer than 2 s or shorter than 200 ms, suggesting lapse of attention, interruption of performance or premature responding, were excluded (∼0.8% trials). For the Control condition, the detection task allowed anticipated responses to the second tone and approximately 25% of RTs fell below the 200-ms criterion. We therefore analyzed the detection RTs with and without applying the low cutoff to check the impact of anticipated responses on the attention effects. For each of the FL and LF conditions, one (different) listener erroneously attended to the irrelevant dimension when incongruent information was presented. However, these listeners performed normally on congruent trials. Their data were excluded from the relevant analyses.

For each TAIL condition, baseline RT was calculated using the trials on which the two tones were the same in both frequency and location. A two (same vs. different frequency) by two (same vs. different location) ANOVA with repeated measures was conducted on the RTs and the error rates. Involuntary orientation was indicated by the impact of the task irrelevant dimension(s) and quantified as the difference between the same and different trials for that dimension. Conflict resolution was indicated by the frequency by location interaction and was quantified as the difference between congruent (same or different in both dimensions) and incongruent (same in one dimension and different in the other) trials (Fig. 1).

#### Auditory processing tasks

We assessed psychoacoustic performance on three tasks: Backward Masking, Simultaneous Masking, and Frequency Discrimination (Fig. 1). Briefly, a 3-interval, 3-alternative forced choice paradigm was used in all of the three tasks (for details, see [5]). At each trial, two identical, standard stimuli and one different, target stimulus were presented in random order. The listeners were asked to report the “odd-one out” by pressing a button. Across trials, the difference between the standard and the target stimuli was adaptively varied following a 3-down/1-up staircase. For each task, two variants were run that yielded different levels of performance. For Backward Masking, the standard stimulus was a 300-ms bandpass noise (600–1400 Hz; 30 dB/Hz). The target stimulus had a 20-ms, 1-kHz tone preceding the noise, with the tone level starting at 90 dB SPL and varied adaptively. In the high-threshold variant (BM0), there was no gap between the tone offset and the noise onset; in the low-threshold variant (BM50), there was a 50-ms silent gap. For Simultaneous Masking, the same bandpass noise and 20-ms tone were used, but the tone started 200 ms after noise onset. In the high-threshold variant (SM), the noise was spectrally continuous; in the low-threshold variant (SMN), there was a spectral notch (800–1200 Hz) around the tone. For Frequency Discrimination, the stimuli were 100-ms tones presented at 75 dB SPL. In the low-threshold variant (FD), the standard tone frequency was fixed at 1 kHz; in the high-threshold variant (FDR), it was roved between 900 and 1100 Hz with a step size of 50 Hz [19].

Frequency Discrimination was administered using the Psychtoolbox for Matlab, with 50 trials per condition. The masking tasks were administered using customized software (IHR-STAR [24]), with 20 trials per condition. Discrimination threshold (‘performance’) at 79% correct was evaluated by fitting psychometric functions using the maximum likelihood method implemented by the Psignifit toolbox for Matlab [25].

## Results

### TAIL Measures

#### Directing attention to frequency

**Figure 2:**
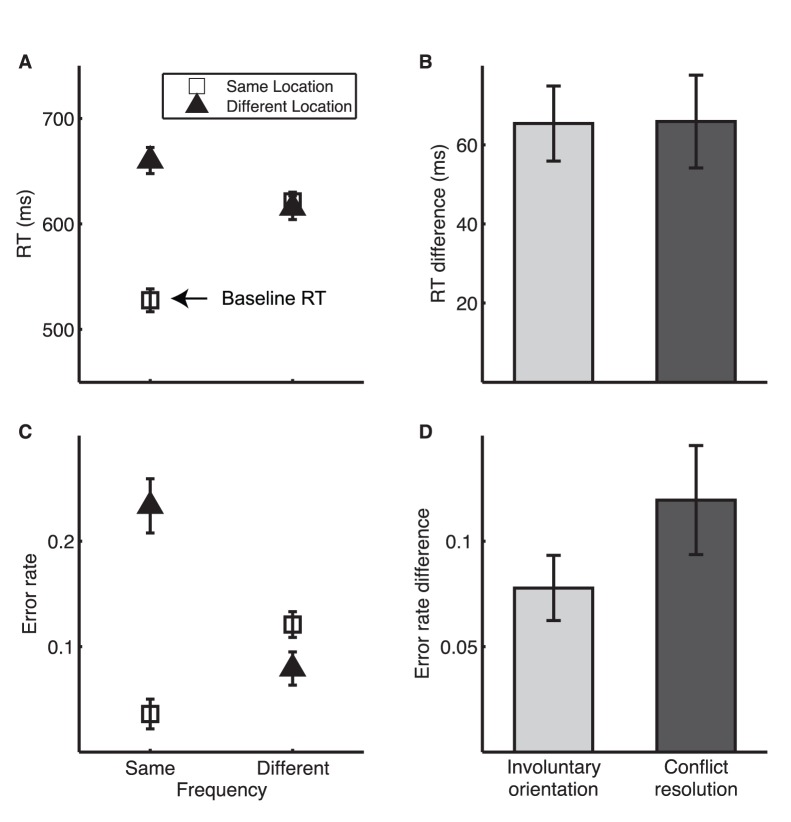
TAIL performance for FL condition. A. Mean reaction time (RT) on correct trials. B. RT difference indicating involuntary orientation to location (RT [different location] – RT [same location]) and conflict resolution (RT [same in one dimension and different in the other] –RT [same or different in both dimensions]). C. Mean error rate. D. Error rate difference indicating involuntary orientation to location and conflict resolution. Here and in the following figures, error bars for RTs are within-subject SEM [50] and error bars for RT differences are across-subject SEM.

In the FL condition of TAIL, RT was shortest when the two tones were the same in both frequency and location (Fig. 2A), which we refer to as baseline RT. We conducted an ANOVA with repeated measures on RT with location (same vs. different ear) and frequency (same vs. different frequency) as within-subject factors. There was no difference between same and different frequencies [F_1,17_ = 2.54, p = 0.13, η_p_^2^ (effect size) = 0.13], indicating approximately balanced processing for the two responses. RT was shorter for the same than for the different location (F_1,17_ = 41.98, p<0.001, η_p_^2^ = 0.71), and shorter when the two dimensions were congruent (same or different in both dimensions, F_1,17_ = 33.60, p<0.001, η_p_^2^ = 0.66) than when they were conflicting (same in one dimension but different in the other, Fig. 1). We thus identified two significant attention effects: **Involuntary orientation** to the task irrelevant dimension quantified as the RT difference between same- and different-location trials (in this FL condition), and **Conflict resolution** as the RT difference between congruent and incongruent trials (Fig. 2B). The involuntary orientation and conflict resolution effects in RT were mirrored by the error patterns (Fig. 2C, D). Error rate was higher for different- than for same-location trials (F_1,17_ = 25.36, p<0.001, η_p_^2^ = 0.60) and for incongruent than for congruent trials (F_1,17_ = 21.35, p<0.001, η_p_^2^ = 0.56).

#### Directing attention to location

**Figure 3:**
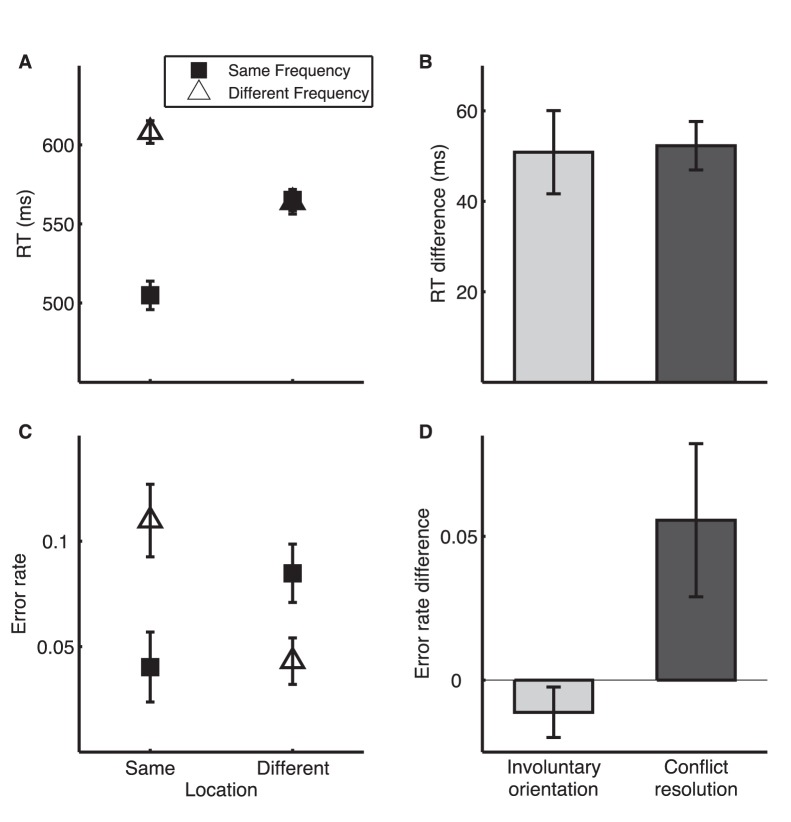
TAIL performance for LF condition. A. Mean RT. B. RT difference indicating involuntary orientation to frequency (RT [different frequency] – RT [same frequency]) and conflict resolution (RT [same in one dimension and different in the other] – RT [same or different in both dimensions]). C. Mean error rate. D. Error rate difference indicating involuntary orientation to frequency and conflict resolution.

We examined whether the attention measures were dimension dependent by running the TAIL in a LF condition in which the task relevant and distracting dimensions were switched. RT analyses showed the same pattern of results as for the FL condition (Fig. 3). RT was longer for different than for same frequency, indicating involuntary orientation of attention to the distracting dimension (F_1,17_ = 30.56, p<0.001, η_p_^2^ = 0.64). Further, RT was longer for incongruent than for congruent trials, indicating a cost of conflict resolution (F_1,17_ = 95.12, p<0.001, η_p_^2^ = 0.85). For error rate, only dimension congruency had a modest but significant effect (F_1,17_ = 4.36, p = 0.05, η_p_^2^ = 0.17), while constancy on the irrelevant frequency dimension did not significantly decrease the likelihood of making an error (F_1,17_ = 2.15, p = 0.16, η_p_^2^ = 0.11).

### Effect of Testing Order

**Figure 4:**
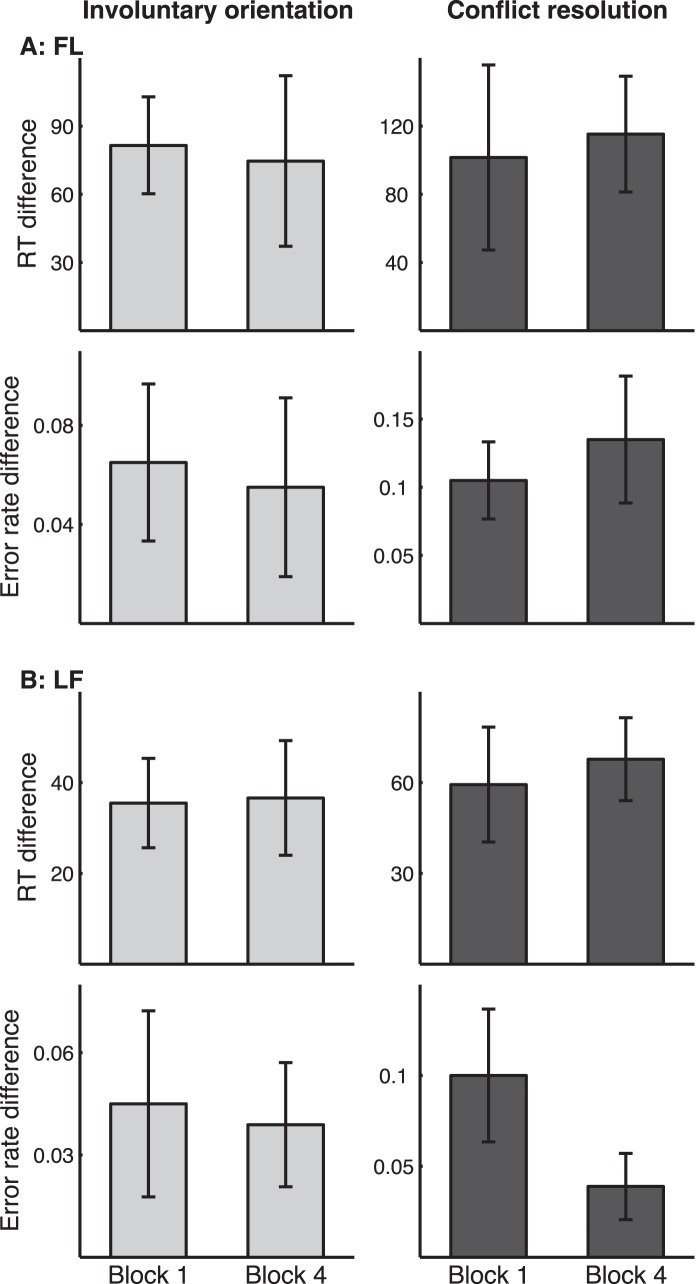
Comparison of RT (row 1 and 3) and error rate (row 2 and 4) measures between the first and fourth blocks of the FL (A, n = 9) and the LF (B, n = 10) conditions in the listeners who performed that condition before the other condition.

We checked whether the attention effects resulted from confusion due to the mixed testing of the FL and LF conditions. If this were true, the effect for each condition would have emerged only after the other condition had been tested. For each condition, we compared the first and the last blocks of trials for those listeners who performed that condition first (Fig. 4). For both involuntary orientation and conflict resolution, RT and error rate differences on the first block were comparable to those on the last block (p>0.2), demonstrating that the effects were not caused by confusion of mixing the conditions. This result also showed the resistance of the attention effects to rapid learning caused by familiarization with the task set and testing environment.

### Attention Effects in a Detection Task

**Figure 5:**
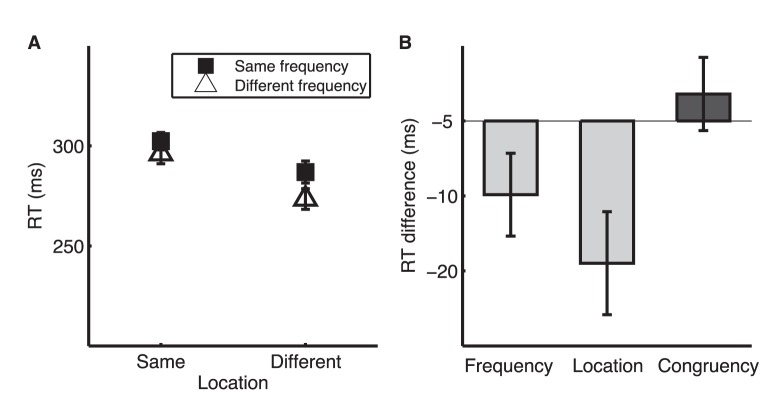
TAIL performance for Control condition. A. Mean RT to the second tone. B. RT gain as a function of frequency constancy, location constancy, and dimension congruency.

We examined whether the attention measures were contingent on the presence of a task relevant dimension by running TAIL in the Control condition, in which the task was simply to detect the second tone and neither frequency nor location was relevant (Fig. 5). RT was significantly shorter for different ear than for same ear presentation (F_1,18_ = 7.75, p = 0.01, η_p_^2^ = 0.30). There was no significant effect of the relative frequency of the tones (F_1,18_ = 3.17, p = 0.09, η_p_^2^ = 0.15) and no frequency by location interaction (F_1,18_ = 0.54, p = 0.47, η_p_^2^ = 0.03). Because the detection task allowed anticipated responses due to the fixed inter-stimulus interval, we repeated these analyses excluding RTs shorter than 200 ms (∼25% of all trials). The results remained the same, with a small significant but negative effect of location constancy (F_1,18_ = 5.39, p = 0.03, η_p_^2^ = 0.23), but no effect of frequency constancy (F_1,18_ = 2.77, p = 0.11, η_p_^2^ = 0.13) or congruency (F_1,18_ = 0.82, p = 0.38, η_p_^2^ = 0.04). The significant negative effect of location constancy indicates that the second tone was processed and impacted responses. The lack of positive effects of feature constancy and dimension congruency suggest that they thus facilitated responses only when a certain stimulus dimension needed to be singled out for judgments.

### Relationship Among TAIL Measures

We examined whether the TAIL measures were correlated, to determine whether they reflected separate functions. Though involuntary orientation and conflict resolution were present when attention was directed to either frequency or location, these measures were not significantly correlated either between or within stimulus dimensions (Table 1). In contrast, baseline RTs were correlated among all three conditions, despite the marked increase from 300 ms in the Control condition to approximately 500 ms in the FL and LF conditions (t>10, p<0.001, Cohen’s d >2.3).

**Table 1:** Correlation between TAIL measures.

R	Orientation(FL)	Conflict (FL)	Orientation(LF)	Conflict (LF)	Baseline (FL)	Baseline (LF)
Conflict (FL)	0.25	−				
Orientation (LF)	0.06	0.27	−			
Conflict (LF)	−0.27	−0.38	0.34	−		
Baseline (FL)	−0.08	0.32	−0.08	−0.24	−	
Baseline (LF)	0.29	0.32	−0.08	0.24	0.82*	−
Baseline (Control)	0.11	0.19	0.23	0.25	0.74*	0.68*

* p<0.001.

### Left Ear Advantage?

Finally, some studies have suggested a left-ear advantage for tonal stimuli [26], [27], [28]. Though any ear advantage should have been controlled for by the counterbalanced presentation of each tone at the two ears, we analyzed if such an advantage was present in the current study. In the LF condition, RT was facilitated when the sounds were presented to the left ear, both in terms of error rate (two ear by two tone position ANOVA, effect of ear, F_1,17_ = 13.9, p = 0.002, η_p_^2^ = 0.69) and RT (F_1,17_ = 35.3, p<0.001, η_p_^2^ = 0.47). This left-ear advantage was greater for the first tone than for the second tone (interaction between ear and tone position, F_1,17_ = 7.6, p = 0.014, η_p_^2^ = 0.34 for error rate, F_1,17_ = 8.4, p = 0.011, η_p_^2^ = 0.32 for RT). A similar but lesser advantage was found in the RT for the Control condition (F_1,17_ = 4.7, p = 0.046, η_p_^2^ = 0.23). However, in the FL condition, the effect of presentation ear on RT switched direction between the first and the second tones (interaction between ear and tone position, F_1,17_ = 8.9, p = 0.009, η_p_^2^ = 0.36), with a left ear advantage for the first tone (follow up t test, t = −2.2, p = 0.042, Cohen’s d = 0.54) but a right ear advantage for the second tone (t = 2.6, p = 0.02, Cohen’s d = 0.63). Thus, the left-ear advantage for tonal stimuli appears to be modulated by attention, with the effect most prominent when attention is directed to location.

### TAIL Predicts Auditory Performance

**Figure 6:**
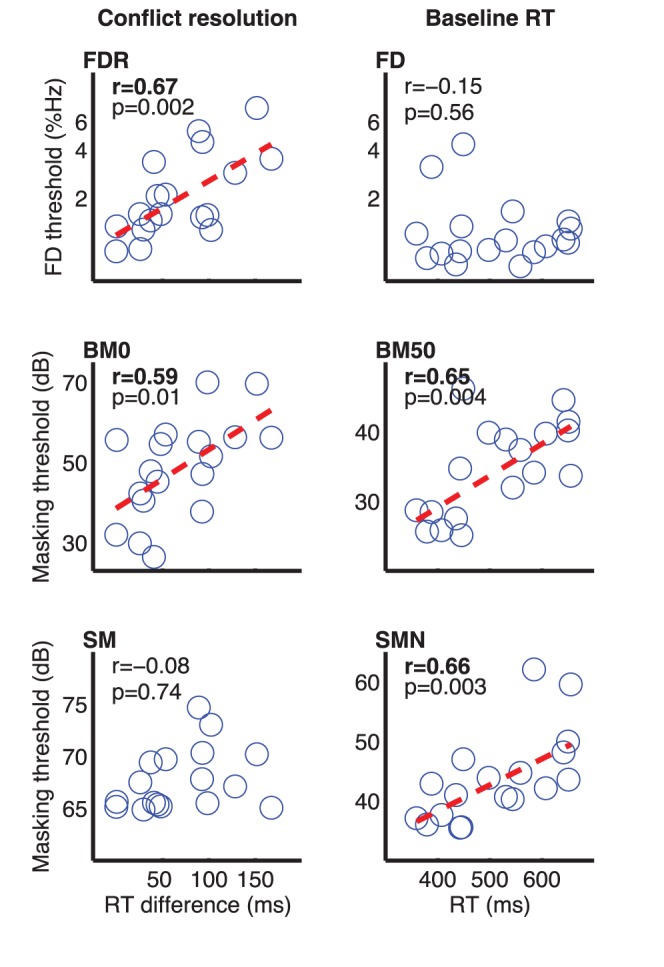
TAIL results predicted auditory perception. Correlations between conflict resolution (left column) and baseline RT (right column) with Frequency Discrimination (top row), Backward Masking (middle row), and Simultaneous Masking (bottom row). A fitted regression line (red dashed) was plotted only for the significant correlations.

A primary motivation of the current study was to develop a test that could be used to assess the contributions of attention to auditory performance. As a first step towards this end, we examined here the extent to which the TAIL measures predicted individual variance in three auditory processing tasks. Among the five independent measures in the FL and LF conditions (orientation to frequency and location, conflict resolution for frequency and location, and baseline RT), only conflict resolution for frequency and baseline RT correlated significantly with threshold performance on the auditory processing tasks tested here (Fig. 6). Conflict resolution accounted for 45% of individual variance on FDR and 35% on BM0, but did not contribute significantly to SM (r = −0.084, p = 0.74) or to the easy conditions of the three tasks (p>0.05). Baseline RTs in the three TAIL conditions, in contrast, accounted for 43–47% of individual variance on BM50 and 43–46% on SMN. Note that all but one of the significant correlations in Fig. 6 would remain so even after a stringent Bonferroni correction for multiple comparisons (at the corrected alpha value of 0.008).

## Discussion

The main goal of this study was to develop a test of attention in listening (TAIL) that could be used to identify contributions of attention to auditory performance. Through manipulation of task relevant and irrelevant dimensions, the TAIL yielded three measures that could be mapped on to the Attention System proposal and the Load Theory of attention as 1) cost of resolving response conflicts, indexing the ability to focus selectively on the relevant dimension and ignore irrelevant ones in decision making, a function of the executive control networks, 2) involuntary orientation of attention to a task irrelevant dimension, reflecting spare perceptual capacity, and 3) response speed in the absence of distracting or conflicting information, indicating efficiency of the information processing system involved. The lack of correlation between the TAIL measures testified to the separation of the attention networks and information processing systems in audition, reminiscent of the Attention Network Test that was designed to test the Attention System view in vision [29]. In addition to integrating multiple facets of attention in one test, we demonstrated how these effects varied with task type (same/different discrimination versus detection) or with different task relevant dimensions (frequency and location). The TAIL measures revealed differential contributions of information processing and attention modulation to threshold performance on an array of psychoacoustic tasks, with executive control accounting for up to 45% of individual differences in some high-threshold variants. To further illustrate the nature of the TAIL measures, we will compare each measure and the associated findings to the relevant attention literature. We will then discuss the implications of the attention contributions to auditory performance revealed by the TAIL.

### Conflict Resolution

The cost of resolving conflicts between sound frequency and location was used to assess the function of the executive control network. A classic example of conflict resolution measure is the Stroop task [17], [18], in which a color name is presented in a physical color that matches or differs from that indicated by the semantic content of the word (e.g., the word ‘red’ presented in red or green font). Naming the color of the word was slower when in the differing (incongruent) than in the matching (congruent) condition, suggesting a cost of resolving the conflict between the two stimulus dimensions. The Stroop effect has been replicated using auditory verbal stimuli, in which the semantic content interfered with perception of pitch or loudness [30], [31]. In vision, conflict resolution has also been demonstrated for non-verbal stimuli. Two widely used examples are the Simon task [32], in which congruency between stimulus location and response direction is manipulated, and the flanker task [29], [33], in which conflicts are introduced in the spatial attributes of target and flanking stimuli.

The TAIL provides a demonstration of non-verbal conflict resolution in the auditory domain. The previous Stroop-like tasks typically measure interference to or along a single stimulus dimension. For example, in the Stroop task, while word content interferes with color naming, there is little interference in the reverse direction. Asymmetric interference between different dimensions like this has been interpreted as reflecting the different degrees of automaticity in processing those dimensions [34]. In the case of the Stroop task, word reading has a more strongly established pathway than color naming in literate people, due to practice and daily use, and would be activated under less executive control (more automatic). In the TAIL, interference was demonstrated to occur in both directions between location and frequency, and to be of similar magnitude on the group level, but uncorrelated on the individual level. The TAIL results thus show that, on the group level, there was no difference in the automaticity of frequency and location processing, but individuals differed in the relative strength of the two pathways. Further, the conflict resolution effect disappeared when the task required simple detection of the second tone, indicating that the recruitment of executive control depends on task demand. Incongruent information in the two stimulus dimensions can be processed without interference and hence without the need for executive control unless the response is contingent on that information. This observation is consistent with the view of executive control as the “top-down” regulating signal conveying task demand [14].

### Involuntary Orientation

According to the Load Theory of attention [16], involuntary orientation of attention to a task irrelevant dimension could be used to index the perceptual capacity spared from processing task relevant information for environment monitoring. While focusing on the task at hand requires exclusion of task irrelevant information, monitoring of the environment for potentially interesting events needs some attention to that information. The Load Theory suggests that, at a fixed cognitive load, attention to irrelevant information is determined by the perceptual capacity spared from task relevant processing. Thus, for a given task and stimulus set, the attention paid to task irrelevant information provides a measure of perceptual capacity: the greater one’s perceptual capacity, the more distraction irrelevant information presents, and the better the ability is to monitor the environment during task performance. An auditory version of the Attention Network Test [31] failed to demonstrate an orientation effect to location as an irrelevant dimension, where the task was to discriminate the pitch of verbal stimuli. In the TAIL, the RT change caused by involuntary orientation to location and frequency was significant and of similar magnitude on the group level suggesting, according to the Load Theory, a significant portion of perceptual capacity being spared. The effect was uncorrelated between frequency and location, suggesting that orienting attention to each dimension involves different mechanisms and/or different pathways. Error rate, however, revealed a notable location advantage on the group level. Erroneous judgments were reduced significantly by location constancy in the FL condition, but not by frequency constancy in the LF condition, from similar levels for cases of inconstancy (9.7% and 9.6% respectively). Thus, frequency constancy speeded the responses to, but did not improve the accuracy of location judgments, while location constancy improved both speed and accuracy for frequency judgments. Errors represent failures of decision making, while RTs reflect sensory processing as well as decision making. The location advantage in accuracy, but not in RT, thus hints at asymmetric depth of processing for frequency and location. Location information appears to be retained and used until a response is made, while frequency information loses influence at an earlier stage of processing. The nature of this asymmetry awaits further investigation, as we are unaware of similar reports or related discussion on this point.

The advantageous effect of location constancy in the FL condition switched to disadvantageous in the Control detection condition. That is, response was slower for same-ear than for opposite-ear presentations. This phenomenon bears a close resemblance to a widely documented attention effect, “inhibition of return” [35], referring to slower responses to a previously cued feature. The inhibitory orienting effects observed here matched or exceeded in magnitude those reported for sound frequency or location [36]. The lack of an effect for frequency was consistent with Mondor et al.’s observation [36] of significant inhibition of return for location, but not for frequency, at a middle stimulus onset synchrony (450 ms; 500 ms on average in the current study). Inhibition of return has been reported to occur later for discrimination than for detection tasks [37], consistent with its absence in the FL and LF conditions. The nature of inhibition of return is still under debate [38], but evidence points to an exogenous (stimulus driven) mechanism that is dissociated from the operation of endogenous (goal driven) attention.

### Baseline RT

Baseline RT (RT in absence of distracting or conflicting information) was intended to assess efficiency of information processing. Consistent with the RT literature [39], baseline RT for the discrimination conditions (FL and LF) was markedly longer than that for simple detection (Control). Despite this difference, baseline RT was highly correlated among all of the three conditions. This correlation conforms to our assumption that baseline RT reflects information processing, which remains largely the same across conditions due to the use of the same stimuli and procedures. The lack of correlation between baseline RT and the derived attention measures (involuntary orientation and conflict resolution) supports the Attention System view of attention and information processing as separate systems [15]. Though baseline RT is not an attention measure, and hence is rarely examined in attention research, it may provide a useful measure in separating contributions of attention from those of information processing and general response mechanisms.

### Relating TAIL to Attention Networks

As the development of TAIL was informed by neuroanatomical attention theories, its outcomes can be speculatively mapped to known attention networks. First, according to the Attention Network view [14], involuntary orienting should be mediated by the frontoparietal orienting network. Corbetta and colleagues [40] divided the orienting network into two parts: a bilateral dorsal network and a right-dominant ventral network. The dorsal network orients attention and modulates sensory processing. The ventral network, inhibited during focused attention, responds to behavioral relevance of unattended stimuli and interrupts current focus of attention for reorientation. In the TAIL, task demand requires participants to direct attention to the target stimulus dimension and to ignore the irrelevant dimension. The involuntary orientation of attention to irrelevant dimensions would reflect inhibition of the ventral network, that is, the suppression of the competition of the irrelevant stimuli for attention. Second, executive control has been associated to anterior cingulate cortex and related brain structures [14]. According to the conflict-monitoring view [22], conflicts in information processing or decision making activates anterior cingulate cortex, which then recruits lateral frontal areas to resolve conflicts. In this framework, the conflict resolution measure of the TAIL would reflect functions of both the conflict monitoring and resolving networks. Another view suggests two separate networks for executive control [41]: a frontoparietal network to initiate control and a cingulo-opercular network to maintain control. In terms of this view, the conflict resolution measure of the TAIL would only capture the function of the cingulo-opercular network, because the measure was obtained during continual task performance. Examining the neural correlates of the TAIL will help illustrate the nature of the attention components measured and fit the test into our current understanding of the neural substrate of attention. It is worth noting that the attention imaging literature is primarily based on visual studies in which attention is oriented to discrete sensory events (for related auditory imaging studies, see [9], [42]). The TAIL instead involves auditory stimuli and manipulation between stimulus dimensions rather than between discrete stimuli. This difference, while a cause for caution when making comparisons, could be informative to the generality of the attention models.

### Attention Contributions to Auditory Perception

Our ultimate goal in developing the TAIL is to evaluate the contributions of attention components to auditory performance. As a first step towards this aim, we correlated the TAIL RT measures with threshold measures on six variants of auditory processing tasks. There are three alternative interpretations for the correlations obtained: 1) attention determines, at least in part, perceptual performance, as we have argued; 2) perceptual performance determines attention; or 3) attention and perceptual performance are both limited by a third factor. Alternative two seems unlikely for two reasons. First, the large differences between stimuli in the TAIL were perceptually highly salient (i.e. easy to distinguish). RT paradigms employing salient stimuli have been widely used to measure attention (e.g., [29], [31], [36]). Second, the measures of attention used in the TAIL were RT differences within conditions with similar stimulus attributes and hence similar bottom-up sensory processing. Any potential perceptual limits to TAIL performance should have been cancelled out. The third alternative, that attention and perception co-vary with a third factor, cannot be easily ruled out, but we are not aware of a plausible third factor that can account for the observed correlation patterns. It could be some other cognitive factor(s) operating in tandem with attention, for example, working memory where executive attention has been hypothesized to play an important role [43]. Disentangling attention components from related cognitive functions is beyond the scope of the current study. A co-varying factor may be neither perceptual nor cognitive, but some other individual variation independent of specific tasks, such as tiredness or anxiety level. However, in such cases, the correlation pattern would be indistinguishable for different measures of perception or attention. While bearing in mind that a list of possible co-varying factors is almost inexhaustible, we suggest that the correlation between TAIL measures and discrimination thresholds likely reflects attention modulation of perception. We will discuss below and in detail how the results fit with the predictions of this interpretation.

The stimuli used in these tasks, pure tones presented either alone or inside a noise, should pose little challenge to perceptual capacity, making involuntary attention unlikely to contribute to task performance. This was what was observed. Contributions of the two remaining TAIL measures, baseline RT and conflict resolution, indicate hierarchical and dimension-specific engagement of executive control in auditory performance. The results are consistent with our prediction of increased executive control in the more challenging task variants, reflecting the greater demand for attention regulation in such conditions. The hierarchical nature was best illustrated in Backward Masking. When the target tone was temporally separated from the masking noise by a 50-ms silence gap (BM50, Fig. 1), perceptual thresholds were predicted by baseline RT, but not by conflict resolution, suggesting that performance was limited by the efficiency of the information processing system, with the executive control mostly inoperative. When the target tone was immediately followed by the masking noise (BM0), the difficulty of separating the task relevant (target tone) from the irrelevant (masker) information increased, and executive control was recruited. This was demonstrated by the significant correlation between BM0 threshold and conflict resolution, but not between BM0 and baseline RT. The contribution of executive control was dimension specific. The ability to focus selectively on frequency, but not the ability to focus on location, was critical. This was consistent with the nature of the stimuli, in which the target and the masker differed in their spectral but not spatial attributes.

The TAIL also revealed different patterns of attention contribution across tasks. For Simultaneous Masking, performance was limited by information processing efficiency when the target tone was spectrally separated from the masker (SMN; Fig. 1). However, when the tone was embedded spectrally and temporally in the noise (SM), performance was not predictable from conflict resolution for either frequency or location. Considering that the spectral difference between the target-present and target-absent noise was very small, a conjecture would be that in SM attention was directed to other dimensions such as loudness. Another possibility is that Simultaneous Masking performance was limited by processes other than conflict resolution. Among the different subtypes of executive control (e.g., shifting, updating, and inhibition [44]), conflict resolution has been modeled to result from regulation of the relative activation of competing processing pathways [45]. The regulation is triggered by conflict detection in anterior cingulate cortex and is done via sensitizing of the target pathway, inhibition of the irrelevant pathway, or a combination of the two. According to this model, for Backward Masking when the target tone is hard to separate from the noise masker (BM0), executive control can enhance performance by simply enhancing stimulus onset responses in the frequency channel from which the onset responses come and/or inhibiting sustained responses in all the other channels. For Simultaneous Masking (SMN), however, there is no clear mark of the temporal or spectral position of the target. The executive control mechanism would be inefficient in distinguishing the competing processes. This account is consistent with functional imaging evidence that anterior cingulate cortex is more active during backward than during simultaneous masking [21]. For Frequency Discrimination, the ability to focus selectively on frequency unsurprisingly predicted performance when the standard tone frequency was roved from trial to trial (FDR). When the standard frequency was fixed (FD), however, performance was predicted by neither executive control nor information processing efficiency. These observations are consistent with the proposal that performance in such circumstances depends on the formation of a memory representation of the repeated standard ('perceptual anchor' [46]).

To our knowledge, this is the first report of auditory performance predicted by an attention test that was administered independently of the perceptual test. The TAIL revealed differential contributions of attention control and information processing across different tasks and different levels of perceptual challenge. In this capacity, the TAIL has potential as a tool for further probing the role of attention in auditory performance. Attention control is a top-down modulating function that is particularly challenged in difficult situations. Thus, the test may be extended for clinical use for separating attention from sensory contributions to impaired perceptual performance. Towards this end, our next step is to examine how TAIL measures relate to everyday auditory performance, including speech comprehension in degraded acoustic environments. Given its simplicity, the TAIL can be used with children or with older adults when presented with an appropriate interface. Children with listening difficulties are often diagnosed with auditory processing disorder, but their listening difficulty might actually result from poor attention rather than from impaired sensory processing [5], [47]. Similarly, the aged population often complains of listening difficulties despite normal audiometric scores [48], [49]. An independent test of auditory attention like the TAIL would help to identify how much those difficulties arise from a declining cognitive control system.

### Conclusion

The TAIL provides individual and quantitative measures of multiple facets of auditory attention and information processing speed, and of the impact of task complexity on those measures. Its use also revealed, within the scope of a limited number of auditory processing tasks, hierarchical and dimension-specific contributions of executive attention to auditory perception. Though the current study is just a first step towards specification and quantification of the contribution of attention to auditory perception, the results have demonstrated the usefulness of the TAIL in achieving this goal and potentially contributing a valid and practical clinical tool.
